# Simplified inducible system for *Trypanosoma brucei*

**DOI:** 10.1371/journal.pone.0205527

**Published:** 2018-10-11

**Authors:** Gabriela T. Niemirowicz, Juan J. Cazzulo, Vanina E. Álvarez, León A. Bouvier

**Affiliations:** Instituto de Investigaciones Biotecnológicas (IIB) Dr. Rodolfo A. Ugalde, Universidad Nacional de San Martín (UNSAM), Consejo Nacional de Investigaciones Científicas y Técnicas (CONICET), San Martín, Buenos Aires, Argentina; Justus Liebig Universitat Giessen, GERMANY

## Abstract

Nowadays, most reverse genetics approaches in *Trypanosoma brucei*, a protozoan parasite of medical and veterinary importance, rely on pre-established cell lines. Consequently, inducible experimentation is reduced to a few laboratory strains. Here we described a new transgene expression system based exclusively on endogenous transcription activities and a minimum set of regulatory components that can easily been adapted to different strains. The p*Tb*FIX vectors are designed to contain the sequence of interest under the control of an inducible rRNA promoter along with a constitutive dicistronic unit encoding a nucleus targeted tetracycline repressor and puromycin resistance genes in a tandem “head-to-tail” configuration. Upon doxycycline induction, the system supports regulatable GFP expression (170 to 400 fold) in both bloodstream and procyclic *T*. *brucei* forms. Furthermore we have adapted the p*Tb*FIX plasmid to perform RNAi experimentation. Lethal phenotypes, including α-tubulin and those corresponding to the enolase and clathrin heavy chain genes, were successfully recapitulated in procyclic and bloodstream parasites thus showing the versatility of this new tool.

## Introduction

Genetic manipulation tools for *Trypanosoma brucei*, a protozoan parasite of medical and veterinary importance, have appropriately been tailored to suit the peculiar regulation of gene expression found in kinetoplastids. In the genome, unrelated protein coding genes are arranged in long units and regulation takes place mainly at the post-transcriptional level [1, 2]. Transcription by RNA polymerase II (RNAPII) produces polycistronic RNA molecules [3, 4] and transcribed intergenic sequences participate in the trans-splicing between the downstream gene and the cap bearing spliced leader, which is transcribed elsewhere in the genome. In addition this processing is also responsible in a concerted manner for the polyadenylation of the upstream coding sequence, thus resulting in the maturation of each mRNA [5, 6]. Furthermore, the remnants of the transcribed intergenic sequence, namely the 5´ and 3´ untranslated regions determine messenger stability and steady state levels as well as translation efficiency through the binding of other cellular components [7–10]. Hence in genetic constructs transgenes are usually placed between intergenic sequences of housekeeping genes. Given this post-transcriptional regulation of gene expression, in principle any transcription activity with enough processivity can be employed in the expression of transgenes. For constitutive expression close to physiological levels, constructs have been inserted into genomic loci to be transcribed by RNAPII readthrough [11, 12]. Alternatively higher expression levels can be obtained placing, into the genetic constructs, any of the available and well-defined RNA polymerase I (RNAPI) promoters. These include those that transcribe the variant surface glycoprotein (VSG) [13] and procyclin acidic repetitive protein (PARP) [14] genes and that are mutually exclusively active in vertebrate and insect specific stages, respectively. On the other hand, transcription irrespective of stage can be obtained with ribosomal RNA (rRNA) promoters [15]. However, since the discovery that bacteriophage RNA polymerases are capable of high processivity transcription in trypanosomes [16] they have been adopted in most regulatable gene expression systems. The arguments supporting this choice involve the fact that T7 RNA polymerase offers reliable high levels of transcription and that unlike other trypanosome transcription activities it is not likely the subject of endogenous regulation [17].

Different approaches for controlling the abundance of gene products have been described. Recently a system based on a drug inducible riboswitch that fused to the mRNA can affect its stability was reported [18]. However, in contrast to the physiological post-transcriptional regulation mechanism, the most widely employed strategy for exogenous control of gene expression involves regulation at the transcriptional level. Endogenous RNAPI or bacteriophage T7 promoters were modified by insertion of bacterial operators enabling accurate control on transcription activity by the drug dependent binding of the corresponding bacterial repressors. Although regulatable expression vectors based on the vanillic [19] and cumate [20] repressors have recently been reported, the most widely used and best characterised inducible systems rely on the tetracycline repressor (tetR) [21].

To date, state of the art inducible systems for trypanosomes can be thought of as being composed of two separate components. The first consists of suitable host strains that have to be established in advance to express the required exogenous regulatory elements. In earlier systems this was accomplished by sequential transfection of two vectors, each one specialized in the constitutive expression of T7 RNA polymerase and bacterial repressor [17, 22]. More recent designs, on the other hand, have managed to express appropriate levels of both these regulatory elements from the same construct and thus the generation of the desired cell line requires a single transfection event [19, 20, 23]. The second component corresponds to vectors in which the sequence of interest is cloned downstream of an inducible promoter. To prevent RNAPII read-through, which would result in higher basal transgene expression, inducible vectors are targeted by homologous recombination to transcriptionally silent regions of the genome. The non-transcribed spacer of ribosomal RNA loci [21] as well as the 177 bp repeats on minichromosomes [24, 25] are two alternative destinations frequently used for this purpose. Finally, for selection of transfected cells these molecules also harbour a constitutively expressed antibiotic resistance gene.

Due to their great success and broad acceptance, inducible systems for *T*. *brucei* have served as models for the development of analogous tools for other kinetoplastid organisms [26–31]. However, there are some drawbacks associated with all of these two component systems. During the establishment of suitable hosts, clonal variability in regulation capabilities has been reported. Usually these differences become manifest only after transfection of an inducible vector, and consequently the following screening steps become laborious [32, 33]. Furthermore, this dependence on pre-establishing cell lines hinders the implementation of inducible system based methodologies in other strains. In this sense, successive transfection and selection steps as well as lengthy periods in culture can affect the natural wild type properties sought after in field isolates.

On the other hand, given the limited number of antibiotic resistance genes available for trypanosome selection, inducible systems that minimize the required number of these elements are highly desirable. To some extent, this issue has been dealt with in works that extend the possibilities for further genetic manipulation methodologies [19, 20, 23]. Moreover, the recent development of an episome-based CRISPR-Cas9 system has allowed genome edition without the insertion of a resistance marker [34], underscoring the interest in methodologies that reduce or circumvent the use of these elements.

In this work we have developed an alternative simplified inducible system based entirely on a single plasmid. It has been designed to contain the sequence of interest along with all other required regulatory elements avoiding the need for pre-established cell lines.

## Materials and methods

### Plasmid construction

For the construction of p*Tb*FIX and p*Tb*FIX-PARP several fragments were PCR-amplified, cloned into pGEM-T Easy (Promega Corporation, Madison, WI, USA) and sequenced (Macrogen, Seoul, Korea). The tetR gene was derived from pLEW13 [17] and the sequence for the c-terminal NLS was added in frame to the 3´ primer. The pLEW100v5 vector (Dr. George Cross Lab, [17]) obtained from Addgene (Cambridge, MA, USA) was used as template for the amplification of the constitutive and inducible versions of the rRNA promoter, as well as the ribosomal spacer targeting sequence and aldolase 3´ intergenic region. Other fragments (pUC min backbone vector, PARP promoter, mEGFP gene and actin and α/β-tubulin intergenic sequences) were derived from already available plasmids. A detailed description of the performed cloning steps can be obtained upon request. The sequences of both vectors were deposited in GenBank under accession numbers MH936667 and MH936668.

Constructs for RNAi gene silencing: For the construction of p*Tb*FIX-αT, Lister 427 genomic DNA was used as template for the PCR-amplification of 550 bp and 864 bp segments from the α-tubulin genes. In each case the same forward (AAGCTT**GCTAGC**CACTACACCATTGGTAAGGAG; HindIII site underlined and NheI site in bold) primer and two alternative reverse primers (GGGCCCGAGACAGAGAGTTGCTCGTGG and GGGCCCTGTGGTCAATACGAGCGAAC; PspOMI site underlined) were used respectively. After cloning into pGEM-T Easy and being sequenced, the HindIII/PspOMI shorter and the NheI/PspOMI longer segments were inserted into HindIII/SpeI digested p*Tb*FIX producing p*Tb*FIX-αT by means of a three fragment ligation. A 1553 bp HindIII/PstI segment from the latter was transferred to p*Tb*FIX-PARP thus giving rise to p*Tb*FIX-PARP-αT. An equivalent strategy was employed for the generation of constructs allowing the RNAi silencing of enolase and clathrin heavy chain genes (p*Tb*FIX-ENO and p*Tb*FIX-CLH). With the same forward primer (AAGCTT**GGATCC**ACGATCCAGAAAGTTCACGGTC; HindIII site underlined and BamHI site in bold) and two reverse primers (TCTAGACCTCAGAACCCATACGCAG and TCTAGAAGCGGCTCATTAATGTCTTTG; XbaI site underlined) 553 bp and 662 bp long fragments from the enolase gene were amplified. Likewise with the forward (AAGCTT**GGATCC**GATTAACCTGAAGCATTCCCAT; HindIII site underlined and BamHI site in bold) and both reverse (TCTAGAACACGTTAATTAGTTTGTCCGCA and TCTAGAGTTCCTGCACCTGCCCAAC; XbaI site underlined) primers 377 bp and 503 bp segments from the CLH gene were cloned. The three fragment ligations into HindIII/BamHI digested p*Tb*FIX involved the shorter HindIII/XbaI fragment with the corresponding BamHI/XbaI longer fragment. All primers used in this work were ordered from Macrogen.

### Trypanosome culture

Pleomorphic bloodstream trypanosome EATRO 1125 cell line (BSF) was cultured at 37°C under 5% CO_2_ in HMI-9 medium (Sigma-Aldrich, St. Louis, MO, USA) [35] supplemented with 20% fetal bovine serum. Lister 427 procyclic cells (PCF) were maintained at 28°C in SDM-79 medium supplemented with 10% fetal bovine serum.

### Generation of transgenic trypanosome cell lines

For each transfection, approximately 2×10^7^ BSF trypanosomes grown to mid log phase (~ 0.8–1.0x10^6^ cells/mL) in HMI-9 medium were collected, washed once with cytomix [36] (2 mM EGTA, 120 mM KCl, 0.15 mM CaCl_2_, 10 mM K_2_HPO_4_/KH_2_PO_4_ pH 7.6, 25 mM HEPES pH 7.6, 5 mM MgCl_2_.6H_2_O) modified with 0.5% glucose, 100 μg/mL BSA and 1 mM hypoxanthine at room temperature, and resuspended in 500 μl of the same buffer. About 5–15 μg of linearized plasmid was mixed with the cell suspension and transferred to a BTX 0.2 cm electroporation cuvette. Electroporation was immediately performed at a single pulse with a BTX 600 Electro Cell Manipulator (BTX Inc., San Diego, CA, USA) at 1.1 kV, 25 μF and R6 resistance (186 Ω). The entire cell-DNA mixture was transferred to a flask containing 5 mL of fresh HMI-9 medium and incubated for 6 hours at 37°C without selection. Cells were diluted to a density of 2x10^5^ cell/mL and 1 mL cell aliquots were distributed in 24-well plates. Selection was achieved by the addition of 0.1 μg/mL of puromycin (InvivoGen).

PCF cells (approximately 2×10^7^ trypanosomes) were washed and resuspended in 500 μl of modified cytomix medium at 4°C. Parasites were electroporated using a single pulse at a voltage of 1.4 kV, 25 μF capacitance and 24 Ω resistance. Selection was achieved by the addition of 1 μg/mL of puromycin.

### Electrophoresis and immunoblotting

Approximately 8x10^6^ BSF or 4x10^6^ PCF cells were resuspended in 1X Laemmli sample buffer, heated in boiling water for 5 min and separated by SDS-PAGE (10 or 12.5% acrylamide). Gels were transferred to a nitrocellulose Hybond ECL membrane (GE Healthcare, Pittsburgh, PA, USA) for probing with anti GFP mouse clones 7.1 and 13.1 antibody diluted 1:1000 (Sigma-Aldrich). Polyclonal rabbit anti enolase was used 1:1000 [37]. Polyclonal anti tetR antibodies were raised in mice (1:500). Monoclonal anti α-tubulin 1:5000 clone B-5-1-2 (Sigma-Aldrich) was used as loading control.

Blots were probed with Alexa Fluor 790 AffiniPure Goat Anti-Mouse IgG (H+L), Alexa Fluor 680 AffiniPure Goat Anti-Mouse IgG (H+L) or Alexa Fluor 680 AffiniPure Goat Anti-Rabbit IgG (H+L) secondary antibodies (Jackson Immunoresearch Laboratories, West Grove, PA, USA). Signal intensities were detected using an Odyssey laser-scanning system and quantified with Image Studio software (LI-COR Biosciences, Lincoln, NE, USA). Prestained Protein Molecular Weight markers used were from Pierce (Rockford, IL, USA).

### Fluorescence microscopy

Cells were fixed with 1% paraformaldehyde, washed with PBS and allowed to bind to poly-L-lysine coated coverslips. GFP fluorescence was monitored with an Eclipse 80i microscope (Nikon, Shinagawa, Japan). 4′,6-diamidino-2-phenylindole (DAPI) was used to visualize the nucleus and kinetoplast.

### Flow cytometry

Mid-log phase density PCF (6x10^6^ cells/mL) or BSF (1x10^6^ cells/mL) cells were analyzed with and without doxycyline (DOX) in a CyFlow space cytometer (Partec, Germany). Subsequent data analysis was performed using the FlowJo VX0.7 software (FlowJo LLC, Ashland, OR USA).

## Results

### Plasmid description

**Fig 1:**
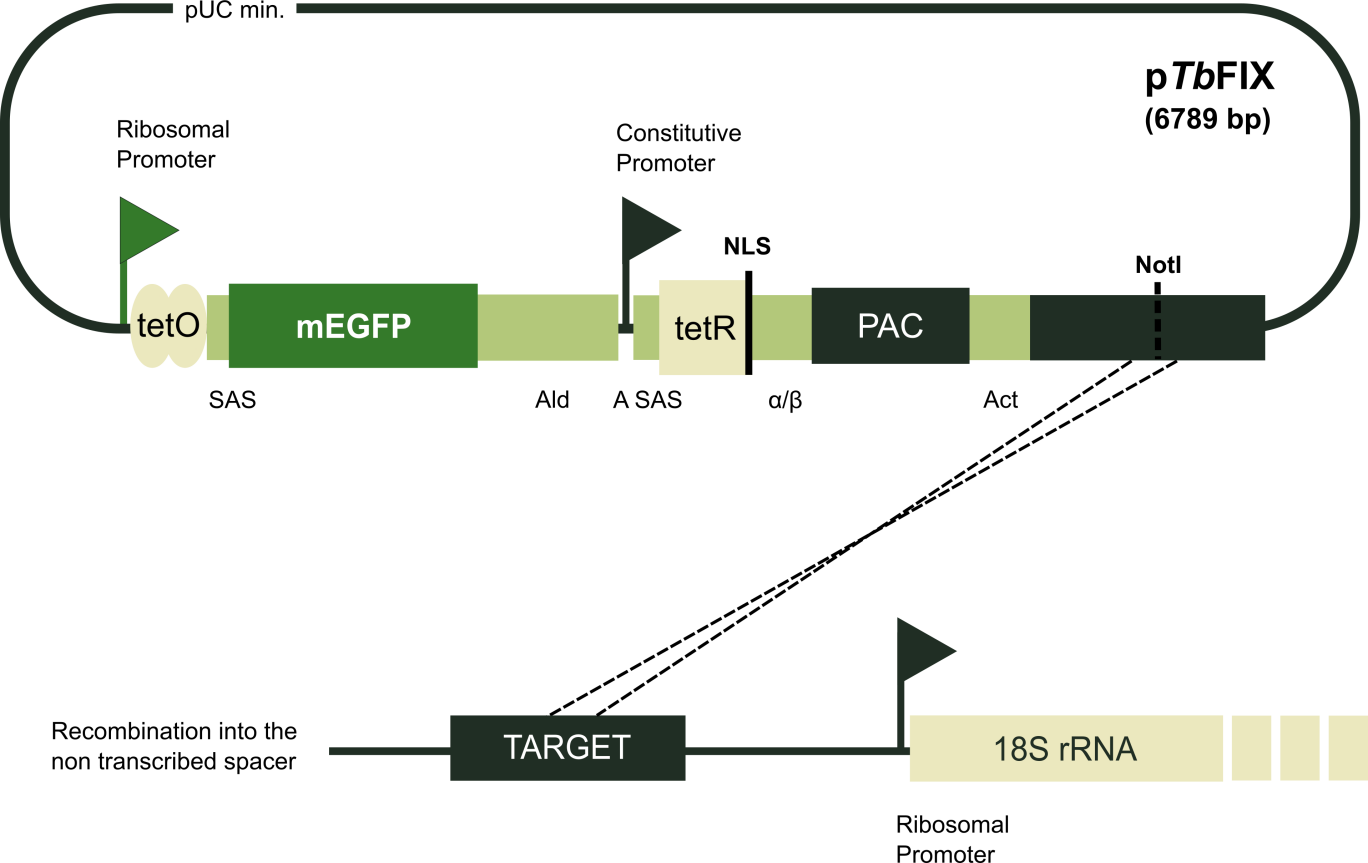
Schematic diagram of p*Tb*FIX vectors. The plasmids contain two transcription units. GFP expression is driven by an inducible rRNA promoter modified to contain 2× tetracycline operators (tetO). The downstream constitutive dicistronic unit encodes the tetracycline repressor (tetR) fused to a nuclear localization signal (NLS) and the puromycin resistance gene (PAC). Constitutive transcription of this unit depends on a second rRNA promoter in p*Tb*FIX or a procyclic acidic repetitive protein promoter in p*Tb*FIX-PARP. The intergenic regions for mRNA maturation are represented by small rectangles: SAS, procyclin splice acceptor site; Ald, aldolase 3´ intergenic region; A SAS, actin splice acceptor site; α/β, tubulin intergenic region; Act, actin intergenic sequence. The p*Tb*FIX vectors are linearized at a unique NotI restriction site that targets the constructs to the non-transcribed spacer of ribosomal RNA gene repeats. Propagation in *E*. *coli* cells is accomplished by a 1.7 kbp long pUC19 derived minimum backbone plasmid (pUC min).

The system conceived for fast inducible expression in *T*. *brucei* is based on vector p*Tb*FIX (Fig 1). The plasmid contains two transcription units. The first drives the expression of a green fluorescent protein (GFP) reporter gene through an inducible rRNA promoter. Downstream, a constitutive dicistronic unit encoding a nucleus targeted tetR and puromycin resistance genes was inserted. To study the effect of different tetR abundances on GFP expression regulation, the original constitutive rRNA promoter in p*Tb*FIX was replaced by that of PARP, thus giving rise to p*Tb*FIX-PARP. After linearization at a unique NotI restriction site inside a targeting sequence, the construct is directed to the non-transcribed spacer of ribosomal RNA gene repeats for integration by single crossover “ends-in” homologous recombination.

### Inducible GFP expression in PCF

**Fig 2:**
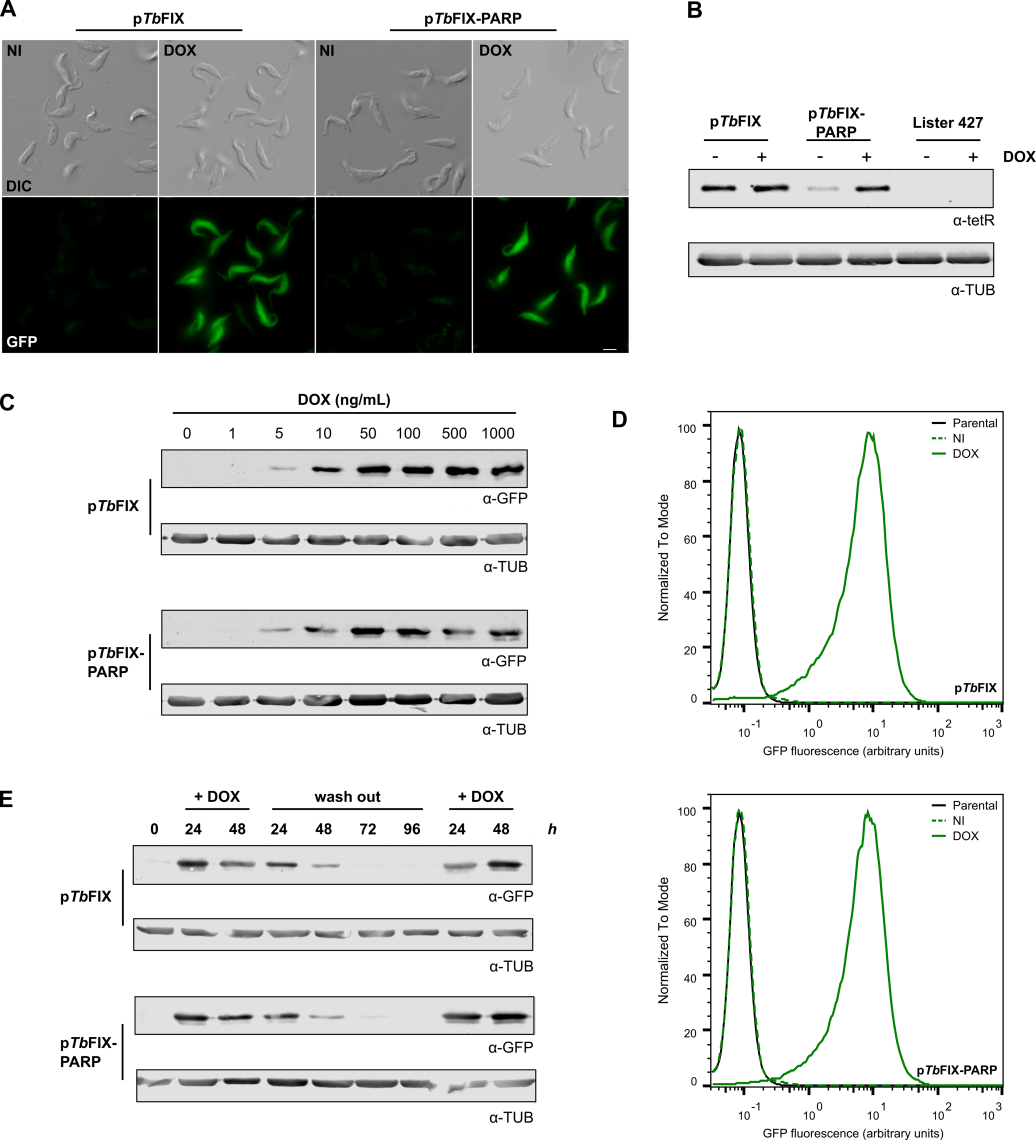
GFP expression in procyclic cells harbouring the p*Tb*FIX vectors. A) Images of Lister 427 p*Tb*FIX and p*Tb*FIX-PARP cells induced for 24 h with 1 μg/mL of DOX. Scale bar 5 μm. B) Western blot probed with anti-tetR antibodies showing the levels of tetR expression in p*Tb*FIX transfected cells (4x10^6^ parasites per lane). For comparative purposes the parental strain (Lister 427) was included in the analysis. C) Doxycycline dose-dependent GFP expression in procyclic cells. GFP expression was measured by Western blot analysis 24 h post induction. D) Fixed induced (DOX) or non-induced parasites (NI) were analysed by flow cytometry. Approximately 50000 events were captured. Mean fluorescence intensity values can be found in S1 Table. E) PCF cultures were diluted to 1x10^6^ cells/mL and induced with 1 μg/mL of DOX. After 48 h parasites were extensively washed with DOX free medium and incubated for additionally 96 h, followed by DOX re-addition. GFP expression levels were monitored daily by Western blot analysis in total cell extracts (4x10^6^ cells per lane). Tubulin was used as a loading control. Cultures were diluted every two days to 1x10^6^/mL to maintain cell density within a range that supports exponential growth.

Both p*Tb*FIX and p*Tb*FIX-PARP vectors where transfected into wild-type Lister 427 procyclic form trypomastigotes and transgenic cell lines were easily obtained. After selection, the resulting parasite populations were cultured with 1 μg/mL of doxycycline which corresponds to the highest concentration employed for other tetR based inducible systems in order to obtain maximal induction [17, 21]. Doubling times of induced and uninduced cells were similar to those of the wild-type parental strain indicating that the constructs had no effect on cellular viability (S1 Fig). Microscopic examination showed a homogeneous population of fluorescent cells in samples derived from induced cultures (Fig 2A). In contrast, no GFP signal could be detected in uninduced equivalents. Protein samples were analyzed by Western blot with antibodies raised against tetR. In uninduced conditions the constitutive rRNA promoter produced higher (4.2-fold) repressor amounts than those obtained with the PARP equivalent. Interestingly, upon doxycycline addition both constructs expressed similar quantities of tetR (1.3-fold higher for p*Tb*FIX). This suggests that the unrepressed inducible rRNA promoter is capable of driving transcription of all three downstream genes (Fig 2B).

In order to determine their regulation properties, transgenic cell lines were grown with different doxycycline concentrations. Western blot analysis showed that both vectors produced similar induction patterns. Abundance of the GFP reporter increased in a dose dependent manner reaching maximum levels with inducer concentrations greater than 50 ng/mL (Fig 2C) similar to that reported for other inducible systems [17, 21, 38]. In these conditions, band quantification indicated ~250 and ~350 fold increase of gene expression in cells transfected respectively with p*Tb*FIX and p*Tb*FIX-PARP.

To confirm these findings and additionally to assess population homogeneity, samples were analyzed by flow cytometry (Fig 2D). In both cases, fluorescence histograms of uninduced transgenic cells overlapped that of the parental wild-type strain indicating tight repression of GFP transcription while addition of doxycycline to 1 μg/mL conduced to a uniform fluorescence increase. These properties were retained even after three months of continuous uninduced culture. In fact, with doxycycline concentrations above 50 ng/mL at least 95% of the cells exhibited fluorescence signal indicating a homogeneous population response (S2 Fig). Within intermediate concentrations the main differences between both cell lines became evident. In the presence of inducer concentrations ranging from 1 to 10 ng/mL, fewer parasites transfected with p*Tb*FIX displayed comparatively lower GFP fluorescence than those transfected with p*Tb*FIX-PARP. This was in agreement with the higher tetR abundance found in the former.

Finally, as can be seen in Fig 2E doxycycline removal can be used to repress transgene expression in these cell lines. In fact GFP abundances decline and reach undetectable levels after four days of culture in inducer free medium. Nevertheless, the fully induced state can be recovered by doxycycline readdition.

### Inducible GFP expression in BSF

**Fig 3:**
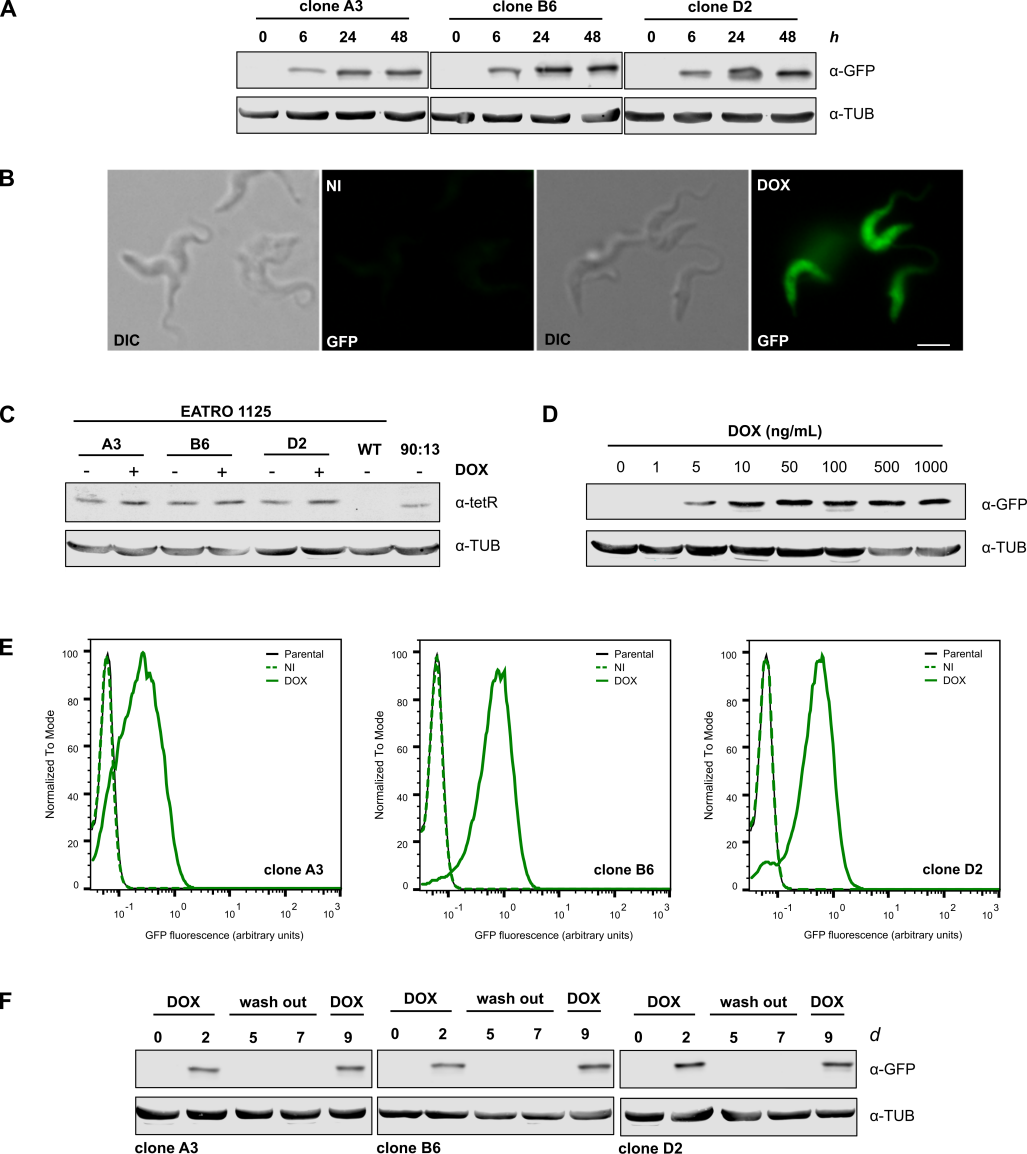
GFP expression in EATRO 1125 cells. A) GFP expression was analysed by Western blot (4x10^6^ cells per lane) in three different clones. Parasites were induced with 1 μg/mL of DOX. Anti-tubulin antibody was used as loading control. B) Images from cultures induced for 24 h with 1 μg/mL of DOX (clone D2). Scale bar 5 μm. C) Western blot probed with anti-tetR antibodies showing the levels of tetR expression in wild type EATRO 1125 cells and three independent p*Tb*FIX clones (8x10^6^ cells per lane). For comparative purposes Lister 427 90:13 parasites were included in the analysis. D) Doxycycline dose-dependent GFP expression in BSF cells. Parasites were diluted to 3x10^4^ cells/mL and induced for 48 h with indicated concentrations of DOX (clone B6). E) Fixed induced (DOX) or non-induced cells (NI) were analysed by flow cytometry. Approximately 50000 events were captured. Mean fluorescence intensity values can be found in S2 Table. E) BSF cultures were diluted to 2x10^4^ cells/mL and induced with 1 μg/mL of DOX. After 48 h parasites were diluted in DOX free medium and incubated for additionally 5 days, followed by DOX re-addition (day 8). GFP expression levels were monitored by Western blot analysis in total cell extracts.

Transfections with both plasmids were performed on wild-type EATRO 1125 strain BSF trypomastigotes. All attempts involving p*Tb*FIX-PARP were unsuccessful, probably due to the downregulation of the procyclin promoter and thus lower expression levels of the puromycin resistance gene. In contrast, viable clones were easily obtained with p*Tb*FIX. Three independent clones were chosen in order to evaluate possible differences in expression patterns and regulation properties. Basal GFP expression levels in all three cell lines were close to undetectable (Fig 3A). However, reporter induction already became evident after six hours of growth with 1 μg/mL of doxycycline. Maximum GFP abundances were reached 24 hours post induction and fluorescence was easily detected by microscopic examination (Fig 3B). No significant deviations of the doubling times were found between induced (~6.4±0.4 h), uninduced (~6.8±0.3 h) and the wild-type parental strain (~6.5±0.5 h) (S3 Fig). However, at this point differences between the clones were already noticed with fold induction levels ranging ~170 (clone A3) and ~400 (clone D2). On the other hand, tetR abundances in the three parasite lines were equivalent and depending on doxycycline presence 1.5-fold or 2.1-fold higher than those in the well-established 90:13 strain (Fig 3C). These results suggested that the upstream inducible rRNA promoter also could contribute to repressor expression in BSF cells.

In this cellular context the system was very sensitive to inducer concentration reaching maximum expression with 10 ng/mL of doxycycline (Fig 3D). On one hand, flow cytometry of these cell lines confirmed tight repression since fluorescence histograms of transgenic uninduced clones overlapped that of the parental wild-type strain (Fig 3E). However, it also revealed additional differences between the clones. Fluorescence of clone A3 was less than that of clone D2. For its part, clone B6 contained the parasites showing the highest fluorescence. Similar to what was found for PCF, induction in the three clones depended on the presence of doxycycline in the medium. Two successive passages in inducer free conditions were enough to fully repress GFP expression, while doxycycline readdition was capable of restoring former expression levels (Fig 3F).

Finally, to asses construct stability these cell lines were kept in culture in an uninduced state for three months with three weekly passages. Induction with different doxycycline concentrations was later analysed by flow cytometry (S4 Fig). Tight repression was retained in all three clones and maximal induction continued to be achieved with doxycycline concentrations above 10 ng/mL. No significant differences were found in population composition of clones A3 and D2, however clone B6 now contained equal amounts of responsive and unresponsive cells.

### System performance in RNAi gene silencing

**Fig 4:**
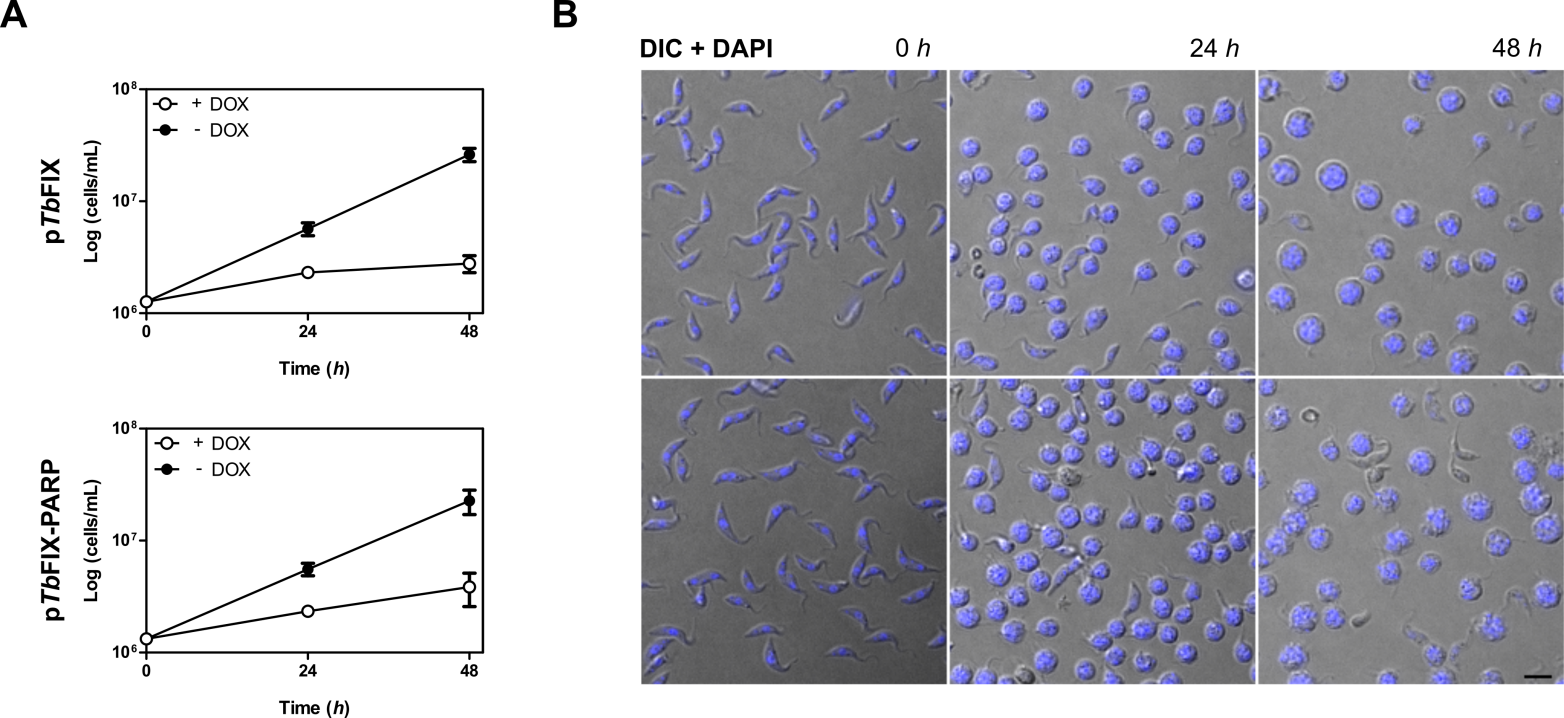
The p*Tb*FIX vectors support RNAi knockdown in PCF cells. A) Growth analysis was carried out in triplicate in cells transfected with the p*Tb*FIX-αT and p*Tb*FIX-PARP-αT plasmids. Values are means ± SD. B) The characteristic FAT phenotype of PCF cell lines expressing α-tubulin dsRNA is visualized by DIC optics after DOX addition (1 μg/mL). Parasite DNA was stained with DAPI. Scale bar 5 μm.

Inducible gene expression systems are frequently adapted for RNA interference mediated endogenous gene downregulation. To test if the vectors developed in this work could be used for this methodology both p*Tb*FIX and p*Tb*FIX-PARP were modified. The GFP coding sequence was replaced with two α-tubulin gene derived segments inserted in opposite orientations. Thus, transcription driven by the inducible rRNA promoter would produce a double stranded RNA molecule in a “stem-loop” configuration. The resulting plasmids (p*Tb*FIX-αT and p*Tb*FIX-PARP-αT) were transfected into Lister 427 PCF cells. No particular difficulties were experienced during the selection period and doubling times of the resulting transgenic lines (12.9±2.5 h and 11.2±0.7 h for p*Tb*FIX-αT and p*Tb*FIX-PARP-αT respectively) were comparable to that of the wild-type parental strain (12.9±0.8 h). However, upon doxycycline addition growth rates declined rapidly indicating impaired cellular viability. The effect was significant with both plasmids but more apparent in cells transfected with p*Tb*FIX-αT 48 hours post induction (Fig 4A). Microscopic examination revealed the reported “FAT” phenotype [39] associated with α-tubulin downregulation in at least 90% of the cells in both cultures (Fig 4B).

**Fig 5:**
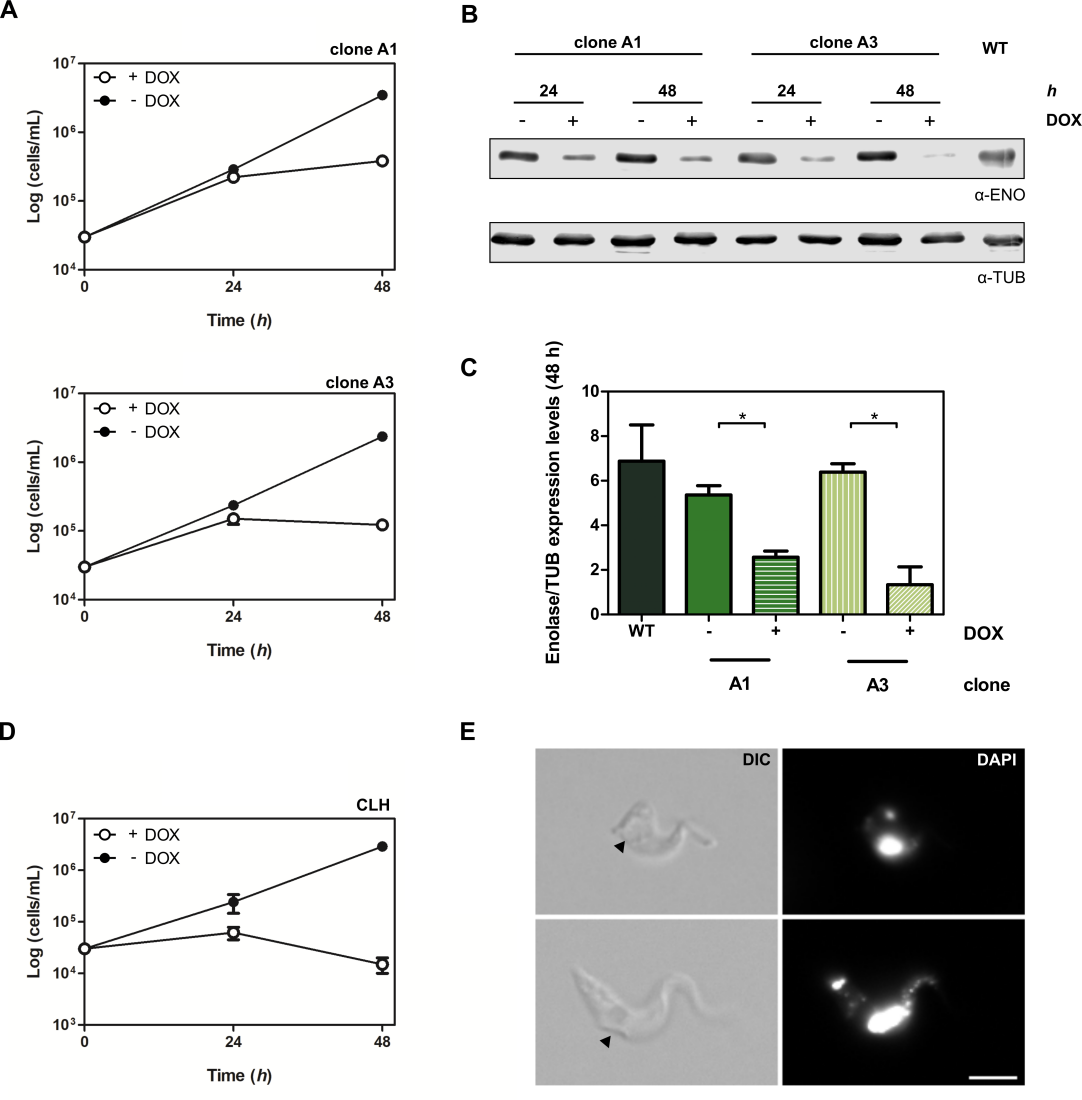
RNAi knockdown in EATRO 1125 BSF cells. A) Growth analysis (triplicate) corresponding to the inducible expression of enolase RNAi in two independent clones. B) Western blot analysis upon enolase RNAi induction. Approximately 5x10^5^ cells were probed with anti-enolase polyclonal antibodies. Tubulin was used as a loading control. C) Enolase quantification signal after DOX addition (1 μg/mL). Values are means ± SD. *P <0.05 (ANOVA). D) Inducible expression of clathrin heavy chain (*Tb*CLH) RNAi in EATRO 1125 BSF cells. Parasite growth was monitored by cell counting in a Neubauer chamber. E) Gallery of images from cultures expressing clathrin RNAi (DOX 1 μg/mL, 24 h). The BigEye morphology is visualized by DIC optics (black arrow). Parasite DNA was stained with DAPI. Scale bar 5 μm.

Regarding BSF, all attempts to transfect p*Tb*FIX-αT into EATRO 1125 parasites were unsuccessful. This limitation might be explained by the already reported sensitivity of BSF even to the slightest basal downregulation of α-tubulin genes [40, 41]. Alternatively, to test if the system could still be used for RNAi silencing of essential genes, two additional p*Tb*FIX derivatives were obtained. With the same RNA “stem-loop” configuration p*Tb*FIX-ENO and p*Tb*FIX-CLH were constructed to target enolase and clathrin heavy chain genes respectively. Given the clonal variability in GFP expression patterns observed for p*Tb*FIX, clones obtained after these transfections were initially screened for loss of viability associated with RNAi induction. In the case of p*Tb*FIX-ENO, no proliferation could be detected in three out of four clones tested with the addition of 1 μg/mL of doxycycline. Two of these cell lines were chosen for further analysis. As can be seen in Fig 5A no growth rate defects could be detected in uninduced conditions. However, 24 hours after RNAi induction proliferation starts to decline and by 48 hours almost no further growth could be detected. Western blot analysis showed differences between the clones regarding enolase abundance (Fig 5B). In uninduced conditions, respective enzyme amounts in clones A1 and A3 were 78% and 93% that of the wild-type parental strain, suggesting tighter RNAi repression in the latter (Fig 5C). After 48 hours of induction values drop to 37% and 19% respectively, explaining the milder growth defects of clone A1.

On the other hand, the initial analysis of two clones obtained for p*Tb*FIX-CLH showed that only one experienced loss of viability when cultured with doxycycline (Fig 5D). Again no effect in proliferation were observed in uninduced conditions, however RNAi induction had even more severe growth defects than those obtained with p*Tb*FIX-ENO. Furthermore, the reported “BigEye” phenotype associated with clathrin heavy chain gene downregulation [42] could be found by microscopic examination of the induced cells (Fig 5E).

## Discussion

This work was focused on the development of a simple genetic manipulation tool that would allow easy implementation of regulated transgene expression in *Trypanosoma brucei*. The main objective consisted in avoiding the dependence on pre-established cell lines, thus easily extending the range of strains on which to apply these methodologies. This could be accomplished with a single DNA molecule containing the sequences of interest as well as those that code for the required exogenous regulatory elements. To keep the design simple, the system was based exclusively on endogenous transcription activities and a minimum set of regulatory components. This was supported by previous works showing that T7 RNA polymerase is not a prerequisite for efficient inducible transgene expression in *T*. *brucei* [24, 43]. Besides, apart from the PAC gene required for selection, the only other exogenous coded element was tetR. The arrangement of inducible and constitutive transcription units in p*Tb*FIX was similar to that of other dual promoter systems [33]. Based on reported poor regulation properties, this configuration was later abandoned in favour of back-to-back alternatives [17]. However, unlike our design, in these pioneering works tetR was expressed from other genomic loci and did not contain a nuclear localization signal which is a feature included in more recent developments [19, 20, 23]. Indeed, the tandem “head-to-tail” configuration was chosen considering that eventual unregulated transcription by the leftmost rRNA promoter in p*Tb*FIX would contribute to tetR expression in a way reminiscent of a negative feedback mechanism. The increase in repressor abundance upon doxycycline addition, most evident in PCF transfected with p*Tb*FIX-PARP, supports this possibility. Finally, during the design phase particular care was taken in order to minimize alterations to the physiological transcription patterns of the cells. In this sense, promoters in p*Tb*FIX vectors and those of the targeted rRNA loci share the same orientation after construct integration.

Transgenic PCF cells experienced equally tight regulation of GFP expression with p*Tb*FIX and p*Tb*FIX-PARP, indicating that tetR levels produced by the former were significantly above those required to repress rRNA transcription. Consequently, further system improvements should be focused on other aspects than increasing repressor amounts. On the other hand, in the fully induced state both constructs reached similar GFP expression levels and cell populations were notably homogeneous. For practical purposes both vectors are equally well suited for conducting regulatable transgene expression based methodologies in PCF cells. However, p*Tb*FIX corresponded to the most versatile tool since it can also be used in BSF. Regulation properties in this cellular background were similar to those observed in PCF. The most apparent difference related to clonal variability, which has already been reported for other constructs [32, 33]. The fact that transgene induction and repression are reversible in all these PCF and BSF lines implies that the system developed can be employed in conditional knockout methodologies.

In principle, p*Tb*FIX has good construct stability properties similar to those reported for other rRNA loci targeted molecules [44]. After three months of continuous culture both PCF cell lines exhibited almost no change in regulation properties and population composition. For their part only one of the three BSF clones experienced loss of GFP expression in a fraction of the cells and, most importantly, all clones retained high repression in uninduced conditions.

It can be speculated that soon after p*Tb*FIX integrates into the genome some unregulated transgene expression might take place until repressor abundances increase. Yet, even with this delay in repression, it was still possible to obtain viable cell lines with constructs for the RNAi downregulation of essential genes in both PCF and BSF parasites. Interestingly, while in uninduced PCF populations obtained with both p*Tb*FIX-αT vectors no cells were found with altered morphology, they showed a noticeably homogeneous response towards induction of RNAi. The former could be due to rapid counter selection of cells with loose repression.

The BSF clonal variability observed not only for GFP expression constructs but also for those intended for RNAi downregulation of enolase as well, can be explained by the already reported position effects associated to alternative site of insertion among different clones [32, 33, 45]. In this respect, besides their use as general purpose transgene expression tools, the p*Tb*FIX vectors might be employed to perform comparative integration site studies in different strains.

Finally, all RNAi constructs in this work were obtained with conventional restriction enzyme based techniques, since these correspond to the most widely available alternative. Nevertheless, p*Tb*FIX vectors can easily be made compatible with other cloning technologies and thus more amenable for high-throughput methodologies [46–48].

## Supporting information

S1 Fig Growth analysis of Lister 427 PCF cells transformed with p*Tb*FIX vectors. PCF cultures were diluted to 1,25x10^6^ cells/mL and induced with 1 μg/mL of DOX. Parasites were counted in a Neubauer chamber every 24 h and diluted every two days to maintain cell density within a range that supports exponential growth. A) Parental strain (Lister 427), B) p*Tb*FIX and C) p*Tb*FIX-PARP cell lines with or without DOX. Doubling times (mean ± SD) are indicated.
(TIFF)

S2 Fig Doxycycline dose-dependent GFP expression in procyclic cells harbouring the p*Tb*FIX vectors. PCF cultures were diluted to 2x10^6^ cells/mL and induced for 24 h with the indicated concentrations of DOX. Flow cytometry analysis was performed in a CyFlow space cytometer. Approximately 50000 events captured per induction. Mean fluorescence intensity values can be found in S1 Table.
(TIFF)

S3 Fig Growth analysis of EATRO 1125 BSF cells transformed with p*Tb*FIX vectors. Parasite cultures, initially diluted to 2x10^4^ cells/mL, were counted in a Neubauer chamber every 24 h. A) For comparative purposes the parental strain (EATRO 1125) was included in the analysis. B, C, D) Growth curves of three independent clones induced or non-induced with DOX (1 μg/mL). Doubling times (mean ± SD) are indicated.
(TIFF)

S4 Fig Doxycycline dose-dependent GFP expression in BSF cells. BSF cultures were diluted to 2x10^4^ cells/mL and induced for 48 h with the indicated concentrations of DOX. Approximately 50000 events captured per induction. Mean fluorescence intensity values can be found in S2 Table.
(TIFF)

S1 Table Arithmetic and geometric mean fluorescence intensity values for procyclic cells harbouring the p*Tb*FIX vectors. (DOCX)

S2 Table Arithmetic and geometric mean fluorescence intensity values for BSF clones. (DOCX)
