# Supplementary figures and images for: Simplified inducible system for Trypanosoma brucei
Source: PLoS One. 2018 Oct 11;13(10):e0205527. doi: 10.1371/journal.pone.0205527 (PMC6181392; doi:10.1371/journal.pone.0205527)

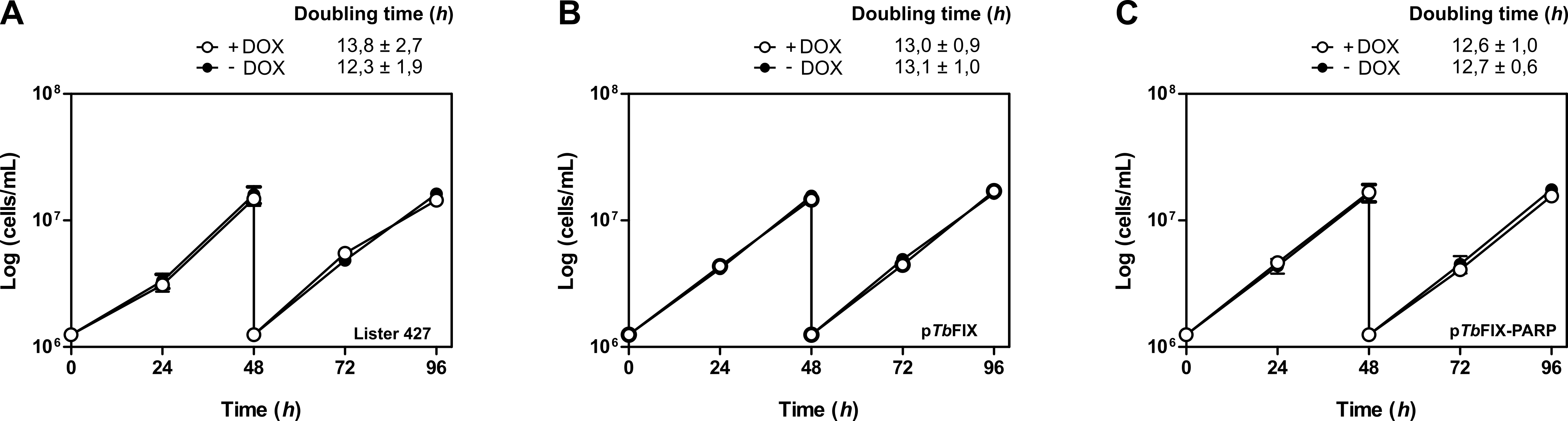

Supplement: S1 Fig — PCF cultures were diluted to 1,25x10^6^ cells/mL and induced with 1 μg/mL of DOX. Parasites were counted in a Neubauer chamber every 24 h and diluted every two days to maintain cell density within a range that supports exponential growth. A) Parental strain (Lister 427), B) pTbFIX and C) pTbFIX-PARP cell lines with or without DOX. Doubling times (mean ± SD) are indicated. (TIFF) [file pone.0205527.s001.tiff]

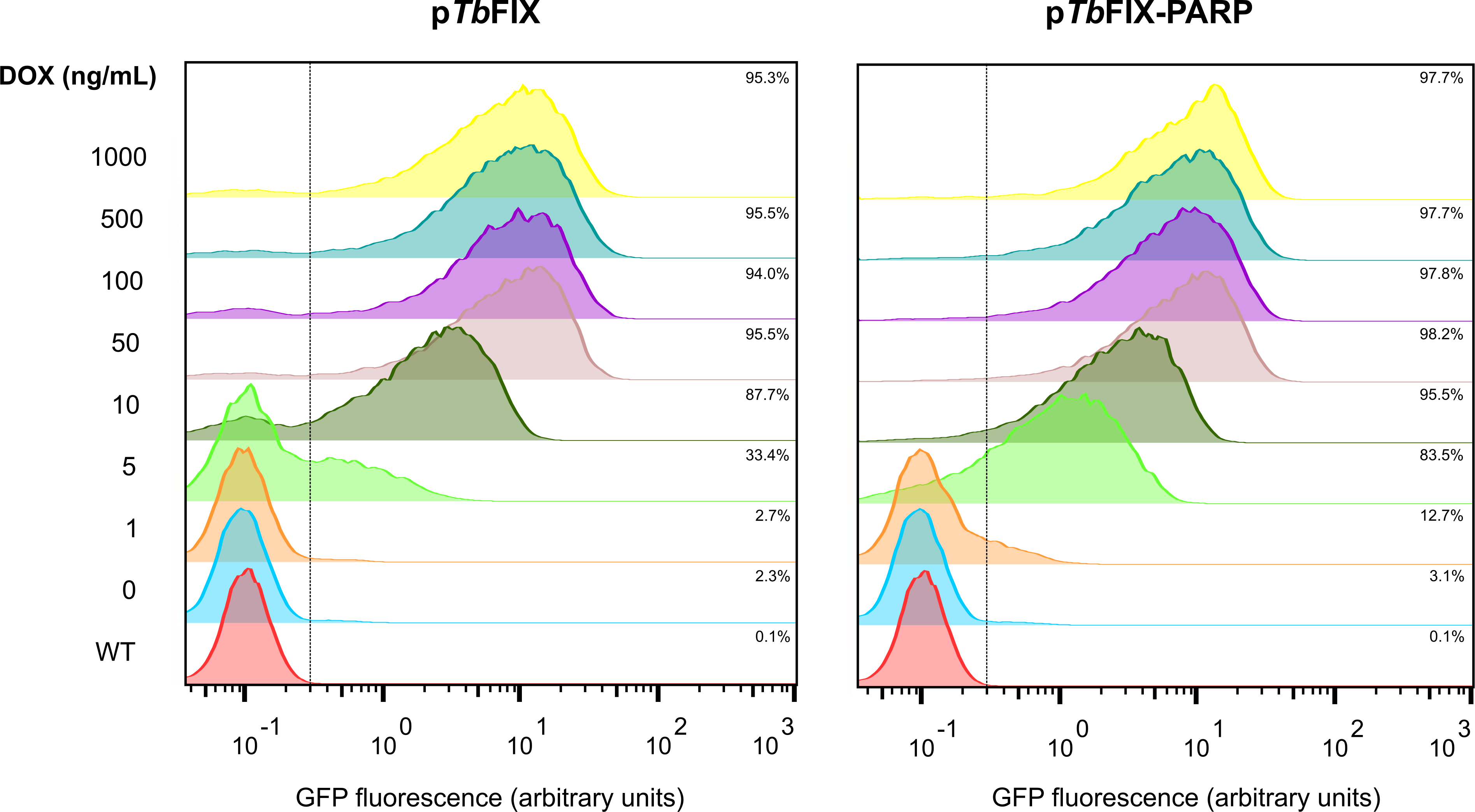

Supplement: S2 Fig — PCF cultures were diluted to 2x10^6^ cells/mL and induced for 24 h with the indicated concentrations of DOX. Flow cytometry analysis was performed in a CyFlow space cytometer. Approximately 50000 events captured per induction. Mean fluorescence intensity values can be found in S1 Table. (TIFF) [file pone.0205527.s002.tiff]

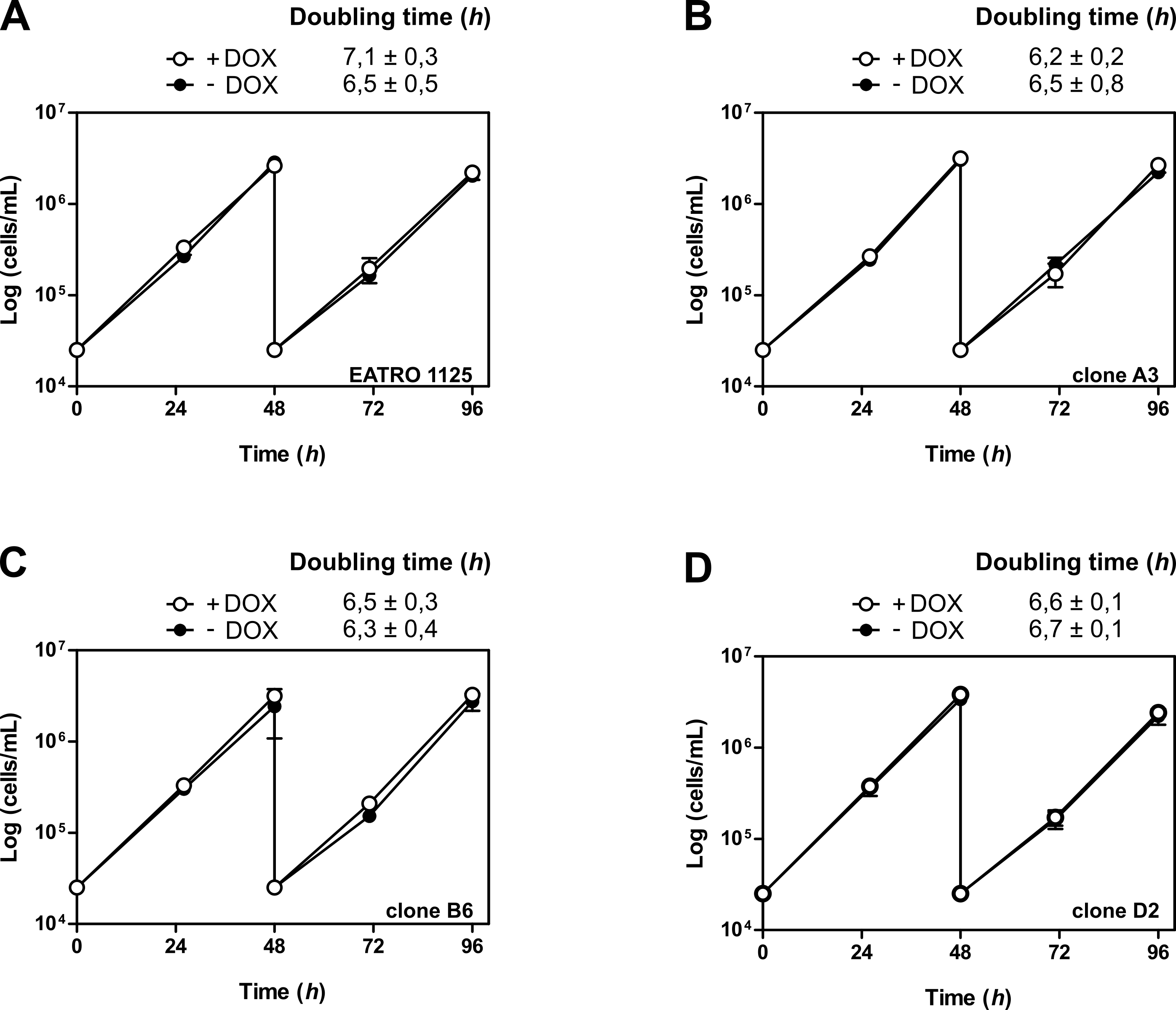

Supplement: S3 Fig — Parasite cultures, initially diluted to 2x10^4^ cells/mL, were counted in a Neubauer chamber every 24 h. A) For comparative purposes the parental strain (EATRO 1125) was included in the analysis. B, C, D) Growth curves of three independent clones induced or non-induced with DOX (1 μg/mL). Doubling times (mean ± SD) are indicated. (TIFF) [file pone.0205527.s003.tiff]

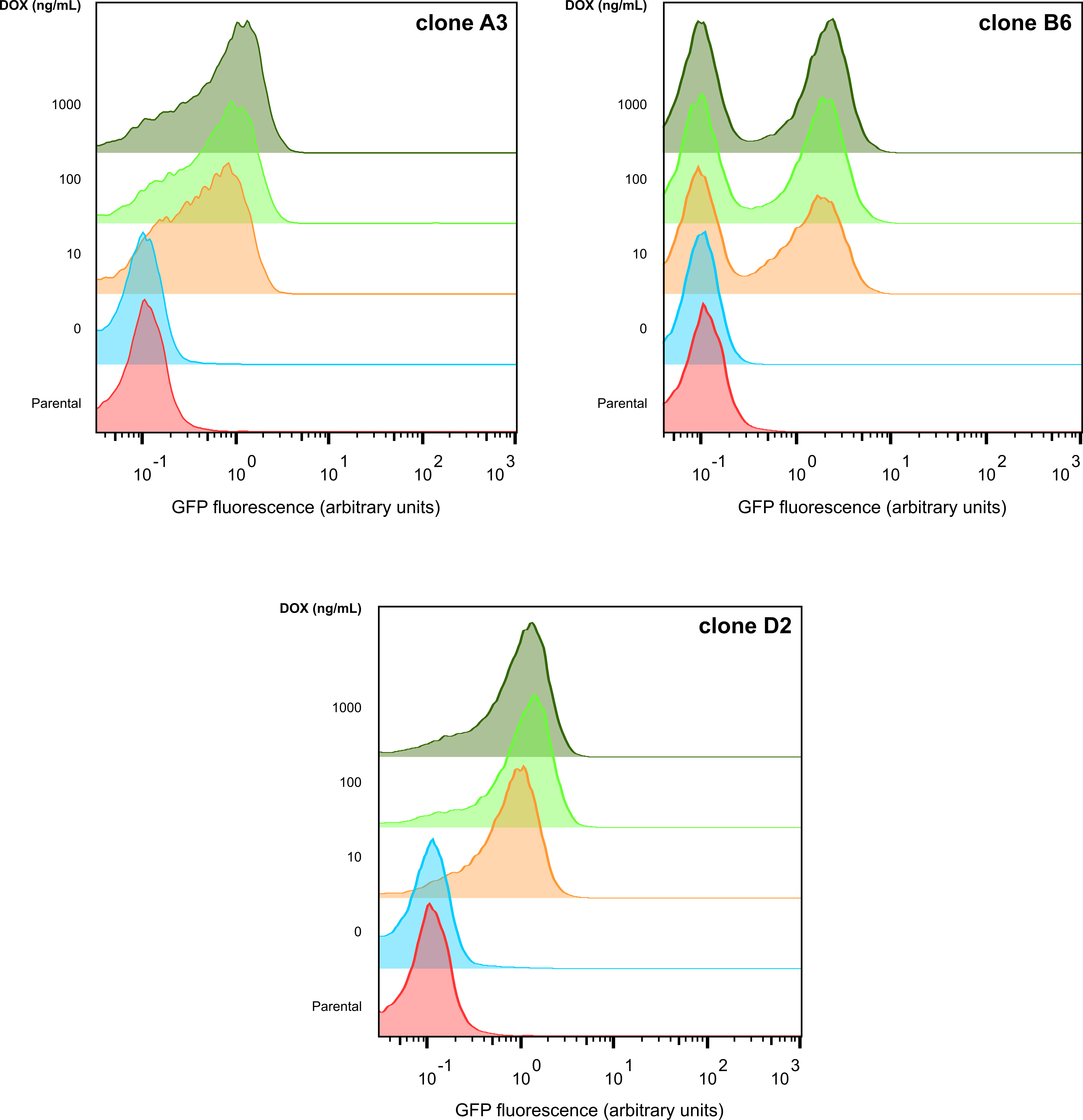

Supplement: S4 Fig — BSF cultures were diluted to 2x10^4^ cells/mL and induced for 48 h with the indicated concentrations of DOX. Approximately 50000 events captured per induction. Mean fluorescence intensity values can be found in S2 Table. (TIFF) [file pone.0205527.s004.tiff]
