# Supplementary material for: Simplified inducible system for Trypanosoma brucei
Source: PLoS One. 2018 Oct 11;13(10):e0205527. doi: 10.1371/journal.pone.0205527 (PMC6181392; doi:10.1371/journal.pone.0205527)
Supplement: S1 Table — (DOCX) [file pone.0205527.s005.docx]

|  |  | | | | | | |  | | |  |  |  |  |
| --- | --- | --- | --- | --- | --- | --- | --- | --- | --- | --- | --- | --- | --- | --- |
|  |  | |  | |  | | |  |  |  |  |  |  |  |
| \|  \|  \|  \| \| --- \| --- \| --- \| | | | | | | |  | |  | | |  |  |  |
| \| **Month** \| **DOX** (ng/mL) \| **p*Tb*FIX** \| \| **p*Tb*FIX-PARP** \| \| **Lister 427** \| \| \| --- \| --- \| --- \| --- \| --- \| --- \| --- \| --- \| \|  \| MFI \| GMFI \| MFI \| GMFI \| MFI \| GMFI \| \| 1 \| 0 \| 0.100 \| 0.090 \| 0.099 \| 0.090 \| 0.092 \| 0.087 \| \|  \| 1000 \| 8.309 \| 5.686 \| 8.443 \| 5.998 \| 0.095 \| 0.088 \| \| 3 \| 0 \| 0.115 \| 0.101 \| 0.120 \| 0.102 \| 0.110 \| 0.103 \| \|  \| 1 \| 0.118 \| 0.102 \| 0.176 \| 0.131 \|  \|  \| \|  \| 5 \| 0.405 \| 0.211 \| 1.369 \| 0.857 \|  \|  \| \|  \| 10 \| 2.728 \| 1.535 \| 3.449 \| 2.318 \|  \|  \| \|  \| 50 \| 9.900 \| 5.889 \| 9.432 \| 6.304 \|  \|  \| \|  \| 100 \| 9.984 \| 5.643 \| 8.730 \| 5.701 \|  \|  \| \|  \| 500 \| 10.05 \| 5.740 \| 8.992 \| 5.803 \|  \|  \| \|  \| 1000 \| 9.796 \| 5.678 \| 10.47 \| 7.116 \|  \|  \| | | | | | | |  | |  |  |  |  |  |  |
|  | | | | | | |  |  |  |  |  |  |  |  |
|  |  |  |  |  |  |  |  |  |  |  |  |  |  |  |
