# Supplementary material for: Simplified inducible system for Trypanosoma brucei
Source: PLoS One. 2018 Oct 11;13(10):e0205527. doi: 10.1371/journal.pone.0205527 (PMC6181392; doi:10.1371/journal.pone.0205527)
Supplement: S2 Table — (DOCX) [file pone.0205527.s006.docx]

| \| **Month** \| **DOX** (ng/mL) \| **Clone A3** \| \| **Clone B6** \| \| **Clone D2** \| \| **EATRO 1125** \| \| \| --- \| --- \| --- \| --- \| --- \| --- \| --- \| --- \| --- \| --- \| \|  \| MFI \| GMFI \| MFI \| GMFI \| MFI \| GMFI \| MFI \| GMFI \| \| 1 \| 0 \| 0.064 \| 0.061 \| 0.066 \| 0.063 \| 0.070 \| 0.064 \| 0.084 \| 0.073 \| \|  \| 1000 \| 0.310 \| 0.229 \| 0.808 \| 0.640 \| 0.569 \| 0.444 \| 0.066 \| 0.063 \| \| 3 \| 0 \| 0.120 \| 0.102 \| 0.109 \| 0.100 \| 0.138 \| 0.110 \| 0.140 \| 0.111 \| \|  \| 10 \| 0.604 \| 0.411 \| 1.177 \| 0.478 \| 0.864 \| 0.656 \|  \|  \| \|  \| 100 \| 0.799 \| 0.550 \| 1.031 \| 0.423 \| 1.194 \| 0.885 \|  \|  \| \|  \| 1000 \| 0.909 \| 0.620 \| 1.279 \| 0.510 \| 1.087 \| 0.810 \|  \|  \| |  |  |  |
| --- | --- | --- | --- | --- | --- | --- | --- | --- | --- | --- | --- | --- | --- | --- | --- | --- | --- | --- | --- | --- | --- | --- | --- | --- | --- | --- | --- | --- | --- | --- | --- | --- | --- | --- | --- | --- | --- | --- | --- | --- | --- | --- | --- | --- | --- | --- | --- | --- | --- | --- | --- | --- | --- | --- | --- | --- | --- | --- | --- | --- | --- | --- | --- | --- | --- | --- | --- | --- | --- | --- | --- | --- | --- | --- | --- | --- | --- | --- | --- | --- | --- | --- |
